# Supra-Annular Transcatheter Aortic Valve Replacement Using Balloon-Expandable Valves in Bicuspid Anatomy With Extra-Large Annuli

**DOI:** 10.1016/j.jaccas.2026.108360

**Published:** 2026-05-19

**Authors:** Hossameldin Hussein, Sudhakar George, Adnan Nadir, Sagar N. Doshi

**Affiliations:** a Department of Cardiology, Queen Elizabeth University Hospital, Birmingham, United Kingdom; b Department of Cardiology, Kasr Al-Ainy Medical School, Cairo University, Cairo, Egypt; c Institute for Cardiovascular Sciences, College of Medical and Dental Sciences, University of Birmingham, Birmingham, United Kingdom

**Keywords:** balloon-expandable valves, bicuspid aortic valve, case series, supra-annular position, large aortic annulus, transcatheter aortic valve implantation

## Abstract

**Background:**

Transcatheter aortic valve replacement (TAVR) in patients with bicuspid aortic valves (BAVs) is challenging. Frequently, heavily calcified BAVs are also commonly associated with annuli exceeding sizing charts for commercial transcatheter heart valves. In many BAVs, supra-annular rather than conventional annular sealing may be feasible when the anatomy is tapered.

**Case Summary:**

We report 3 patients with BAV and very large annuli who underwent TAVR, in which successful outcomes were achieved using balloon-expandable valves (BEVs), with sealing and anchoring within the bicuspid leaflets rather than the annulus. In all cases, a transcatheter heart valve considerably smaller than the annulus was used successfully. Despite supra-annular positioning, coronary access was not compromised, and absence of migration was confirmed with interval multislice computed tomography.

**Take-Home Messages:**

Supra-annular TAVR can be successfully performed in patients with BAV and a very large annulus when the anatomy is tapered. Good anchoring and sealing can be achieved within the leaflets. Supra annular TAVR with short-framed BEVs may preserve coronary access better than taller framed self-expanding valves.

**Visual Summary:**
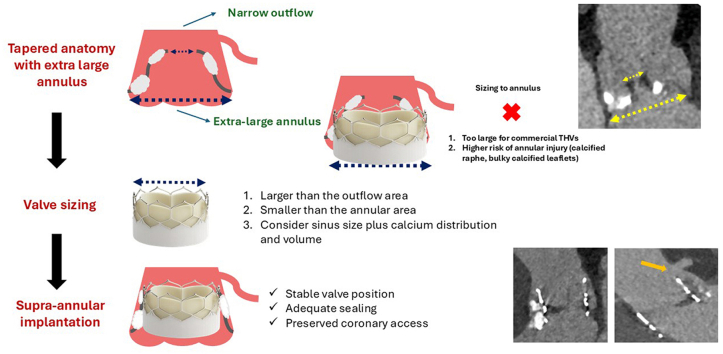
Supra-Annular TAVR With Edwards Sapien 3 Device in Tapered Bicuspid Valve Anatomy TAVR = transcatheter aortic valve replacement; THV = transcatheter heart valve.

Conventional sizing of transcatheter heart valves (THVs) in transcatheter aortic valve replacement (TAVR) has relied on measurement of the aortic valve annulus. The sizing charts for all commercial THVs are based on the annular perimeter/area. TAVR in bicuspid aortic valves (BAVs) carries an increased procedural risk, particularly in patients with heavy leaflet or raphe calcification.^1^^,^^2^ Expert consensus continues to support THV sizing using standard annular dimensions in BAV.^3^

**Take-Home Messages**

• TAVR can be successfully performed in patients with bicuspid aortic valves despite a large annulus when the outflow is narrow, as seen in a “tapered anatomy.”
• Supra-annular sealing can be achieved with balloon-expandable devices.
• Balloon aortic valvuloplasty with simultaneous aortography can help guide sizing and reduces the risk of paravalvular leak.

BAVs often coexist with large annuli, exceeding the size range of available THVs. However, BAV anatomy is frequently tapered, whereby the outflow at the free edge of the leaflet has a smaller perimeter than the annulus, which may allow supra-annular rather than conventional annular sealing to occur. Supra-annular sizing methods such as LIRA (Level of Implantation at the RAphe)^4^ and CASPER (Calcium Algorithm Sizing for bicusPid Evaluation with Raphe)^5^ are well described, however they apply only to self-expandable valves. For balloon-expandable valves (BEVs), 2 approaches—the intercommissural distance^6^ and the circle method^7^—have been proposed. However, no clear guidance on THV positioning has been provided with the intercommissural distance method, and the circle method has not been clinically validated.

We present 3 patients with BAV with extra-large annuli (>683 mm^2^) and tapered anatomy who were treated with the Edwards Sapien 3 BEV and supra-annular sealing using a novel, reproducible method of sizing and positioning.

## Case Presentation

### Patient 1

A 44-year-old man presented with heart failure due to severe aortic stenosis of a BAV. At age 13, he had undergone balloon aortic valvuloplasty but was then lost to follow-up. Despite optimal medical therapy and cardiac resynchronization therapy with defibrillator, he remained symptomatic and was referred for consideration of cardiac transplantation.

Transthoracic echocardiography (TTE) showed a severely dilated and impaired left ventricle (LV) (ejection fraction: 10%). The maximum aortic valve velocity (AVmax) was 2.9 m/s, and the aortic valve area index (AVAi) was 0.5 cm^2^/m^2^. Cardiopulmonary exercise testing showed a Vo_2_ max of 60 mL/kg/min (50% for his age), and the patient was deemed too well for cardiac transplantation. The heart team consensus was to perform TAVR.

Multislice computed tomography (MSCT) showed a type 1 BAV. The annulus measured 1,084 mm^2^ and was too large for any commercial THV. Anatomy was tapered, with an outflow area of 91 mm^2^. The left main and right coronary heights were 24 mm and 18.5 mm, respectively, with no significant coronary artery disease (Figure 1).

**Figure 1:**
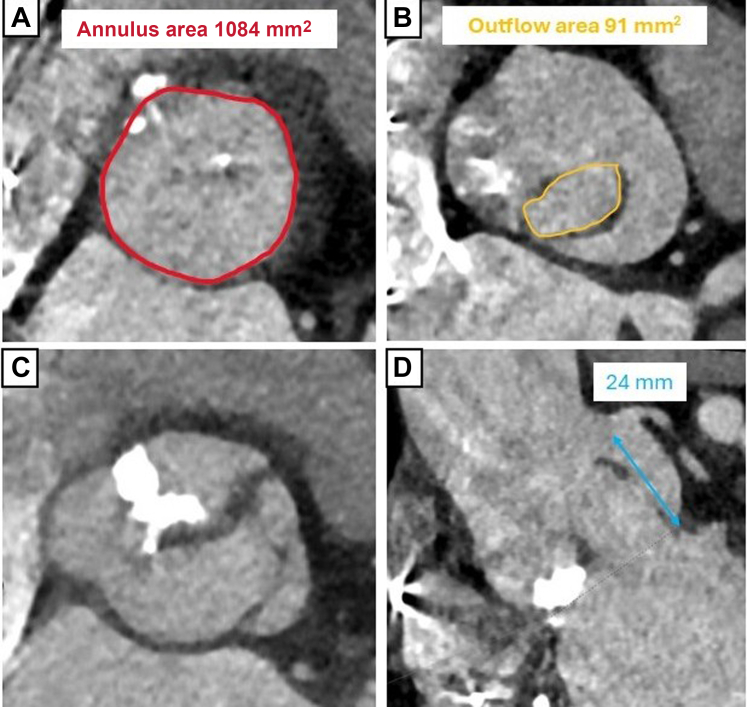
Patient 1: Pre-TAVR Computed Tomography Aortography (A) A large aortic annulus with a perimeter of 117 mm and area of 1,084 mm^2^. (B) Tapered anatomy with outflow area of 91 mm^2^. (C) Bicuspid leaflets with heavily calcified raphe between fused right and noncoronary cusps. Sinus of Valsalva size: 39.1 × 51.7 mm. (D) A high origin of the coronary left main stem at 24 mm. TAVR = transcatheter aortic valve replacement.

Supra-annular TAVR with a 29-mm Sapien 3 valve was planned. The sinus of Valsalva was large enough to accommodate a valve area of ∼900 mm^2^. Overfilling of a 29-mm Sapien 3 Resilia by 8 mL was planned to achieve an area of 850 to 900 mm^2^, thereby improving the effective orifice area (EOA) and supporting LV recovery.

The THV was positioned with the lower frame on the annular plane, with slow deployment under burst pacing (Figure 2A, Video 1). Post-TAVR aortography showed the bottom of the THV to be wholly supra-annular, with trivial aortic regurgitation (Video 2). There were no complications, and the patient was discharged uneventfully the following day.

**Figure 2:**
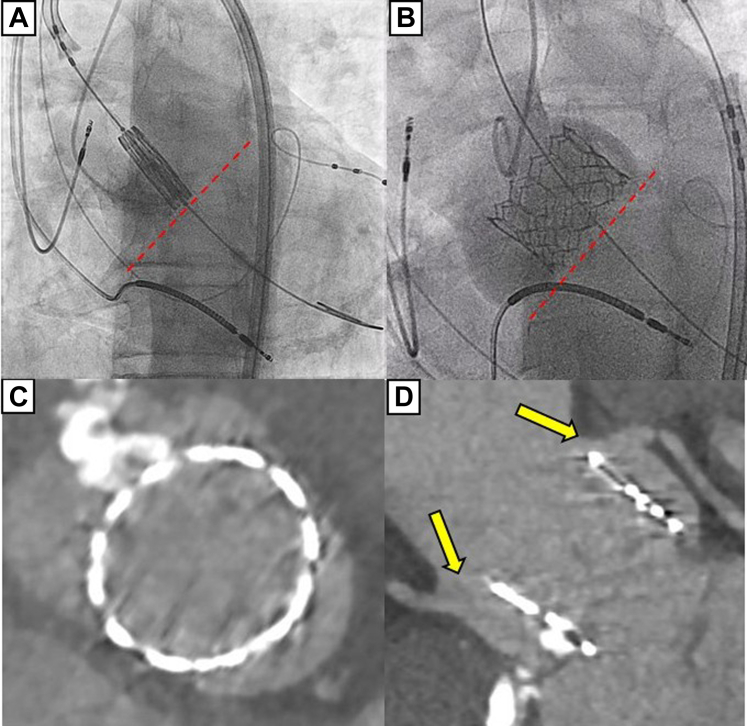
Patient 1: Post-TAVR Aortography and Multislice Computed Tomography (A) Sapien 3 positioned resting on annular plane at deployment (red dotted line). (B) Postdeployment aortography showing the Sapien 3 deployed above annulus. (C) Post-TAVR MSCT, axial cut at the level of the dilated sinus of Valsalva, showing mild asymmetry but good frame expansion. (D) Post-TAVR MSCT, longitudinal cut, confirming access to both coronary ostia outside the Sapien 3 frame (arrows). MSCT = multislice computed tomography; TAVR = transcatheter aortic valve replacement.

MSCT at 1 month confirmed supra-annular positioning, no migration, and good coronary access outside the THV frame. There was mild asymmetry at the level of calcified raphe, but overall good frame expansion (Figures 2C and 2D, Video 3). At 3 months, the patient reported symptom improvement with no cardiac readmissions. TTE showed an LV ejection fraction of 45%, AVmax of 1.16 m/s, mean gradient of 3 mm Hg, and no paravalvular leak.

### Patient 2

A 72-year-old man presented acutely with heart failure symptoms. TTE showed severe LV impairment (ejection fraction: 10%). The AVmax was 3.49 m/s and the AVAi was 0.27 cm^2^/m^2^. The heart team consensus was to perform TAVR.

Gated MSCT showed extreme calcification of a type 2 BAV. The annulus was large, with an area of 691 mm^2^, and the BAV was tapered, with an outflow area of 97 mm^2^. The left and right coronary heights were 15.2 mm and 15.7 mm, respectively (Figure 3).

**Figure 3:**
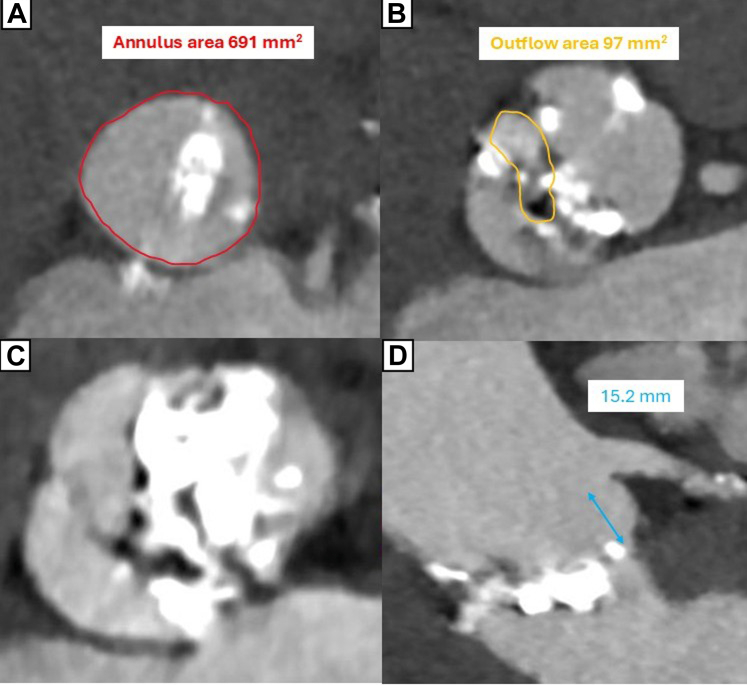
Patient 2: Pre-TAVR Computed Tomography Aortography (A) A calcific aortic annulus with a perimeter of 93 mm and area of 691 mm^2^. (B) Outflow area of 97 mm^2^. (C) Heavily calcified raphe between fused right and left coronary cusps. Sinus of Valsalva size: 32.9 × 39.6 mm. (D) Coronary left main stem height at 15.2 mm. TAVR = transcatheter aortic valve replacement.

Because of the very bulky calcification, conventional sizing to the annulus with a 1-mL overfilled 29-mm Sapien 3 was felt to carry a high risk of sinus rupture. Supra-annular placement of an underfilled 29-mm Sapien 3 by 1 to 2 mL was considered but was rejected on the grounds of associated “pinwheeling” and its connection with premature structural valve degeneration. Furthermore, a 1- to 2-mL underfilled 29-mm Sapien 3 was still considered to carry an increased risk of sinus rupture, given the extreme and bulky calcification. Supra-annular TAVR with a nominally filled 26-mm Sapien 3 was deemed the safest strategy. Predilatation with a 25-mm semicompliant Cristal balloon (Balt) was performed. Aortography at full balloon inflation showed no paraballoon leak, confirming that adequate sealing would be achieved with a 26-mm Sapien 3 (Figures 4A and 4B, Video 4).

**Figure 4:**
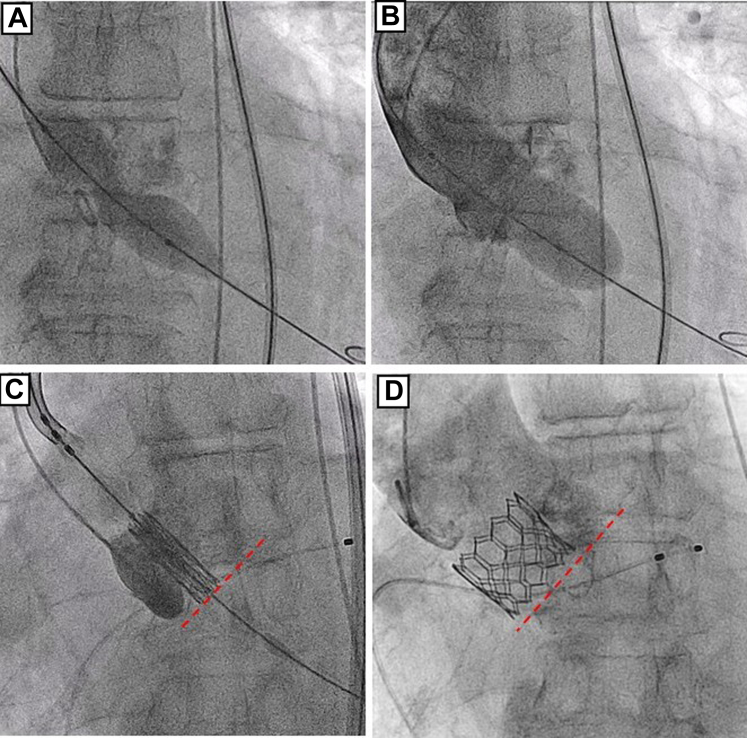
Patient 2: Aortography at Predilatation, Full Balloon Expansion, and Postdeployment (A) Predilatation with a 25-mm Cristal balloon showing a significant waist due to heavy calcification. (B) Full balloon expansion with no evidence of paraballoon leakage on aortography. (C) Sapien 3 positioned on annular plane at deployment (red dotted line). (D) Postdeployment aortography showing a well-seated valve in the supra-annular position.

At deployment, the 26-mm Sapien 3 Resilia was positioned with the frame resting on the annular plane and was deployed with burst pacing (Figures 4C and 4D, Video 5). There was trivial aortic regurgitation on aortography, with good access to the left coronary artery.

TTE demonstrated considerable improvement in LV function, with ejection fraction increasing to 45%. AVmax was 2.41 m/s, mean gradient 13 mm Hg with mild paravalvular leak. Twelve-lead electrocardiogram showed new left bundle branch block.

Gated MSCT at 1 week confirmed a stable position, with mild asymmetry at the midframe opposite the heavily calcified raphe and good access to both coronary arteries outside the frame (Figure 5).

**Figure 5:**
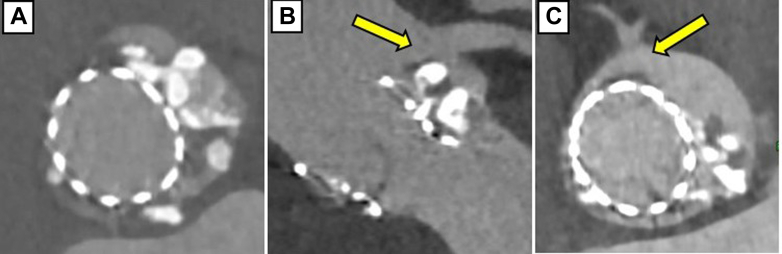
Patient 2: Post-TAVR Multislice Computed Tomography (A) Axial cut at the level of the sinus showing a well-expanded valve with mild asymmetry due to high calcium burden. (B) Longitudinal cut demonstrating unobstructed access to the left main stem outside the Sapien 3 frame (arrow). (C) Axial cut showing good access to the right coronary artery outside the THV frame (arrow). TAVR = transcatheter aortic valve replacement; THV = transcatheter heart valve.

### Patient 3

A 66-year-old man presented with syncope from ventricular tachycardia while driving. He reported a 4-month history of progressive exertional dyspnea. TTE showed a poorly functioning LV (ejection fraction: 10%-15%), an AVmax of 2.94 m/s, mean gradient 19.3 mm Hg, and aortic valve area of 0.85 cm^2^.

Gated MSCT showed a heavily calcified, type 1 BAV. The annulus was extra large, with a perimeter-derived area of 1,267 mm^2^. The left and right coronary heights were 18.4 mm and 20.2 mm, respectively, with no significant coronary artery disease. The anatomy was tapered, with an outflow area of 86 mm^2^ (Figure 6).

**Figure 6:**
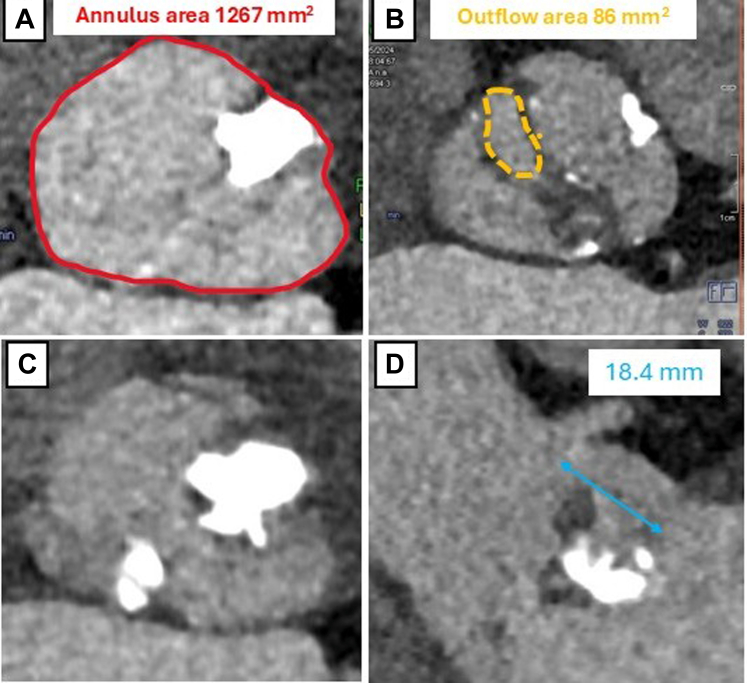
Patient 3: Pre-TAVR Computed Tomography Aortography (A) A calcific aortic annulus with a perimeter of 126 mm and area of 1,267 mm^2^. (B) Tapered anatomy with outflow area of 86 mm^2^. (C) Heavily calcified raphe between fused right and left coronary cusps. Sinus of Valsalva size: 39.6 × 44.9 mm. (D) Coronary left main stem height at 18.4 mm. TAVR = transcatheter aortic valve replacement.

TAVR was recommended by the heart team. The extremely large annulus exceeded all commercial THV sizes. Given the tapered anatomy, supra-annular positioning and sealing with a BEV was considered optimal. Although a nominally filled 29-mm Sapien 3 would have provided good anchoring and sealing within the leaflets, overfilling by 8 mL to achieve a larger EOA to maximize LV recovery was deemed optimal.

Under conscious sedation, a 29-mm Edwards Sapien 3 Resilia was overfilled by 8 mL. Without predilatation, the valve was positioned with the lower frame on the annular plane at deployment (Figures 7A and 7B, Video 6). Aortography showed no aortic regurgitation, and the left main coronary artery remained accessible above the frame (Video 7). There was no conduction disturbance, however the patient received cardiac resynchronization therapy with defibrillator given poor LV function and syncope with documented ventricular tachycardia. TTE at 18 months showed a stable THV. The AVmax was 2.3 m/s and mean gradient was 15.5 mm Hg, with no paravalvular leak. Ejection fraction improved to 35%. The patient remained clinically well, with no heart failure readmissions. MSCT at 5 months showed a stable THV with uniform expansion and good access to the dominant left coronary artery (Figures 7C and 7D, Video 8).

**Figure 7:**
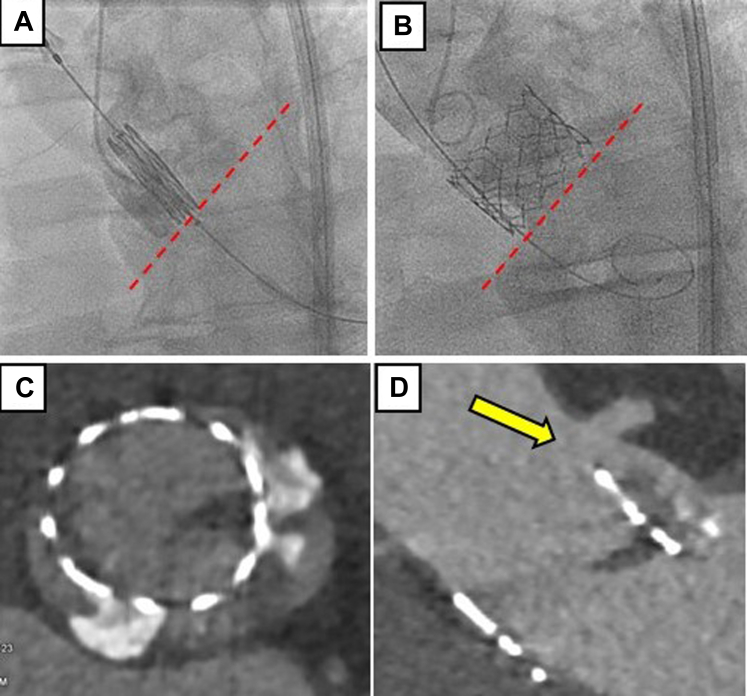
Patient 3: Post-TAVR Aortography and Multislice Computed Tomography (A) Sapien 3 positioned on annular plane at deployment (red dotted line). (B) Post-TAVR aortography showing the Sapien 3 frame above the annular plane (100%-0% level). (C) Post-TAVR MSCT, axial section at the level of sinus, showing good expansion and circularity. (D) Post-TAVR MSCT, longitudinal section, showing free access to the left main stem ostium outside the Sapien 3 frame (arrow). MSCT = multislice computed tomography; TAVR = transcatheter aortic valve replacement.

## Discussion

BAVs frequently exhibit a “tapered” configuration whereby the outflow at the free edge of the leaflet typically has a perimeter smaller than that measured at the virtual annulus. Sealing and anchoring of THV prostheses may therefore occur within the tapered zone, between the virtual annulus and the outflow. Multiple observational studies have proved this concept, showing that a THV smaller than the virtual annulus can result in good clinical and procedural outcomes.^4^, ^5^, ^6^

Four different sizing methods have been proposed as an alternative to conventional annular sizing, based on the presence of tapered anatomy, allowing supra-annular sealing and anchoring. As yet, there is no agreement as to which of these is preferred, and systematic comparisons between these strategies are currently lacking. The LIRA method^4^^,^^8^ was validated as a sizing strategy exclusively for self-expandable valves in type 1 and 2 BAV. In this method, the perimeter at the level of the maximal protrusion of the raphe (LIRA plane), obtained by tracing the internal borders of the leaflets, is measured in addition to the perimeter of the annulus. The smaller of the 2 measurements is used to choose prosthesis size, which in tapered anatomy will correspond to that at the LIRA plane. In the BAVARD registry study, the intercommissural distance,^6^ measured 4 mm above the annulus, was used to select the appropriate THV diameter; results using either self-expanding (Lotus/Evolut R) or balloon-expandable (Sapien 3) platforms showed similar outcomes to patients with tricuspid anatomies. In the CASPER method,^5^ an algorithm based on calcium score, raphe length, extent of calcification and the annular perimeter is used to select prosthesis size. The CASPER method was studied only in self-expandable valves and has not been assessed in BEV-treated patients. In contrast to the previous methods, the semiquantitative circle method^7^ applies to the Sapien 3 BEV. In this method, circles with a THV diameter one size smaller than that predicted by the annulus are projected 3, 6 and 9 mm above the annular plane. If the circle is large enough to touch the commissures, then sealing is anticipated.

There is limited guidance on Sapien 3 positioning for supra-annular sealing in patients with BAVs. In the BAVARD registry study, no specific guidance was given for valve positioning using the intercommissural distance.^6^ The circle method recommends placing the central marker of the valve 3 mm above the annular plane or aligning the annulus with the radiolucent line to achieve a final position 90% to 10% to 100% to 0% aortic/ventricular.^7^ To date, no systematic study has been undertaken to validate the circle method, and it remains largely a theoretical concept.

In the presented case series, a simplified method of device size selection was based on the perimeter of the outflow at the free edge of the leaflet, while considering the annular and sinus of Valsalva dimensions. All 3 patients demonstrated a markedly smaller outflow perimeter compared to annulus. Accordingly, a THV was selected with a perimeter larger than the outflow but smaller than the annulus, with additional consideration of sinus size as well as the bulkiness and distribution of leaflet calcium. A summary of the anatomical measurements and THV sizes for the 3 patients is shown in Table 1.

**Table 1:** Comparative Summary of Anatomical Measurements and THV Sizing Across the 3 Cases

	Patient 1	Patient 2	Patient 3
Annular area, mm^2^	1,084	691	1,267
Outflow area, mm^2^	91	97	86
Sinus of Valsalva dimensions, mm	39.1 × 51.7	32.9 × 39.6	39.6 × 44.9
THV size selection	29-mm S3 + 8 mL	26-mm S3 + nominal filling	29-mm S3 + 8 mL
Estimated THV area, mm^2^	849	519	849
Undersizing % relative to annular area	−22%	−25%	−33%
Oversizing % relative to outflow area	+833%	+435%	+887%

S3 = Sapien 3; THV = transcatheter heart valve.

In 2 cases (patients 1 and 3), substantial overfilling of a 29-mm prosthesis (8 mL) was undertaken. Both patients had sufficient sinus space to accommodate a prosthesis with an area of 850 to 900 mm^2^, and given severely impaired ventricular function, maximizing the EOA was prioritized to aid LV recovery. Nevertheless, in both cases adequate sealing and anchoring would have been achieved with a nominally filled device. In patient 2, there was concern that a 29-mm prosthesis may cause sinus injury given the bulky leaflet and raphe calcification. Therefore, a nominally filled 26-mm prosthesis was chosen. Balloon aortic valvuloplasty using a 25-mm semicompliant balloon with simultaneous aortography proved helpful in confirming good sealing before prosthesis deployment.

As demonstrated in all 3 patients, the THV was positioned with the lower frame resting on the virtual annulus at deployment. The 26-mm and 29-mm Sapien 3 shorten on deployment by 7 mm and 8.5 mm, respectively, with the majority of foreshortening occurring in the lower frame half, anticipating that the lower frame edge would land considerably above the annulus on full deployment. In all cases, postprocedural MSCT confirmed the THV position above the virtual annulus, indicating that sealing and anchoring occurred exclusively within the bicuspid leaflets. There was trivial aortic regurgitation in two of the cases and none in the third.

### Practical Tips for Supra-Annular THV Implantation

1. Start with the lower frame border on the annular plane. In contrast to conventional Sapien 3 positioning, where the lower border of the 3-mm central marker on the deployment balloon is positioned on the annular plane, supra-annular deployment is achieved by positioning the *lower border* of the Sapien 3 *frame* on the virtual annulus, allowing the natural foreshortening of the device (predominantly in the lower half) to lift the prosthesis into a fully supra-annular final position. This provides predictable landing above the annulus.
2. Use balloon valvuloplasty and aortography to confirm sealing. Valvuloplasty with a semicompliant balloon and simultaneous aortography may help confirm whether the supra-annular outflow zone can achieve adequate sealing with the intended THV size. This was particularly valuable in patient 2, where a 25-mm balloon demonstrated no paraballoon leak, supporting the choice of a nominally filled 26-mm Sapien 3.
3. Maintain a controlled, slow deployment under burst pacing. Slow deployment under burst pacing is essential and allows the operator to make any minor adjustment to positioning as necessary.

## Conclusions

Our cases introduce new insights for supra-annular TAVR in patients with BAV whose annular dimensions fall outside the Sapien 3 device sizing chart. The suggested sizing method is simple and incorporates measurement of the outflow perimeter at the free edge of the leaflet as well as the size of the sinus of Valsalva, together with the distribution and bulkiness of leaflet calcification. As demonstrated, sealing and stability were achieved in all patients, with no migration on interval MSCT. In addition, coronary access was preserved outside the stent frame, indicating that redo TAVR could be achieved without the need for leaflet modification. Further research is still required to explore the long-term outcomes of supra-annular TAVR, with more focus on challenges for redo procedures.

## Funding Support and Author Disclosures

Prof Doshi is a proctor for Edwards Lifesciences. All other have reported that they have no relationships relevant to the contents of this paper to disclose.

## Appendix

Video 1 Patient 1: The THV Is Positioned With the Lower Frame on the Annular Plane, With Slow Deployment Under Burst Pacing

Video 2 Patient 1: Post-TAVR Aortography Showing the Bottom of the THV to be Wholly Supra-Annular With Trivial Aortic Regurgitation

Video 3 Patient 1: MSCT at 1 Month Showing Supra-Annular THV Positioning, Good Coronary Access Outside the THV Frame, and Overall Good Frame Expansion

Video 4 Patient 2: Predilatation With a 25-mm Semicompliant Balloon, With Simultaneous Aortography at Full Balloon Inflation Showing No Paraballoon Leak

Video 5 Patient 2: The 26-mm Sapien 3 Resilia Is Positioned With the Frame Resting on the Annular Plane and Deployed With Burst Pacing

Video 6 Patient 3: The THV Is Positioned With the Lower Frame on the Annular Plane at Deployment

Video 7 Patient 3: Aortography Shows No Aortic Regurgitation, and the Left Main Coronary Artery Remains Accessible Above the Frame

Video 8 Patient 3: MSCT at 5 Months Showing a Stable THV With Uniform Expansion and Good Access to the Dominant Left Coronary Artery
